# TRIM14 recruits BRCC3 to stabilize PD-L1 by suppressing p62-dependent selective autophagy in glioma

**DOI:** 10.3389/fimmu.2026.1942769

**Published:** 2026-10-13

**Authors:** Yan Wu, Xujun Wang, Mengqiu Li, Rong Wang, Yaoxing Wu, Di Liu

**Affiliations:** 1 Institute of Precision Medicine, the First Affiliated Hospital, Sun Yat-sen University, Guangzhou, Guangdong, China; 2 MOE Key Laboratory of Gene Function and Regulation, State Key Laboratory of Biocontrol, School of Life Sciences, Sun Yat-sen University, Guangzhou, Guangdong, China; 3 Department of Critical Care Medicine, The First Affiliated Hospital of Sun Yat-sen University, Sun Yat-sen University, Guangzhou, Guangdong, China; 4 Organ Transplant Center, The First Affiliated Hospital, Sun Yat-sen University, Guangzhou, China

**Keywords:** glioma, PD-L1, selective autophagy, TRIM14, ubiquitination

## Abstract

Glioma remains largely refractory to immune-checkpoint blockade, in part because tumor-intrinsic mechanisms sustain programmed death-ligand 1 (PD-L1) and reinforce an immunosuppressive microenvironment. Tripartite motif-containing 14 (TRIM14) is an immune associated protein. However, the role of TRIM14 in PD-L1 homeostasis in glioma is incompletely defined. Here we reported that TRIM14 was increased in malignancies and was particularly associated with overall survival in glioma. Single-nucleus analysis showed that TRIM14-positive nuclei were predominantly malignant, indicating association between TRIM14 and glioma progression. We also found TRIM14 upregulated PD-L1 protein levels without materially changing *CD274* mRNA in glioma cells. Additionally, knockout of TRIM14 reduced tumor growth *in vivo*. Mechanistically, TRIM14 recruits deubiquitinase BRCC3 to reduce K63-linked ubiquitination of PD-L1 in glioma cells, thereby inhibiting p62-mediated autophagic degradation of PD-L1. This pathway prompts that TRIM14 or BRCC3 maybe candidate targets for therapeutic intervention in glioma.

## 1 Introduction

Diffuse gliomas, particularly glioblastoma, are among the most lethal primary tumors of the central nervous system (1). Molecular classification and multimodal treatment have improved diagnostic precision and disease control, yet durable remission remains uncommon because of infiltrative growth, cellular plasticity, treatment resistance, and profound local immunosuppression (2, 3). Immune-checkpoint blockade has transformed the treatment of several extracranial malignancies, but the clinical activity in glioblastoma has yielded limited benefit (4, 5). These reports indicate the necessity to define mechanisms that regulate immune checkpoint abundance, and thereby shape the probability of antitumor immunity.

Programmed death ligand 1 (PD-L1, encoded by *CD274*) suppresses T cell activation through engagement of programmed cell death protein 1 (PD-1) (6). Beyond transcriptional induction by interferon and oncogenic signaling, PD-L1 protein levels are also controlled by trafficking, proteasomal or autophagic degradation (7–9). Post-translational control is essential for biological process requiring rapid and reversible immune adaptation. In glioma, a checkpoint-stabilizing mechanism that is shared across malignant states could have broad consequences for immune escape (10).

TRIM14 is a member of the tripartite motif family that lacks a canonical really interesting new gene (RING) structural domain, and functions primarily as a signaling and protein-interaction scaffold (11). Previous work has connected TRIM14 to innate immune signaling, selective autophagy, inflammatory transcription, and glioma chemoresistance (12–14).

Here, we combined bulk and single-nucleus patient-data analyses with mechanistic experiments to determine the function of TRIM14 in glioma. We showed that TRIM14 was broadly expressed across malignant glioma states, and post-transcriptionally stabilized PD-L1. We further identified BRCC3 as the deubiquitinase recruited by TRIM14, which was required for removal of K63-linked ubiquitin and blockade of p62-mediated selective autophagy. Our findings support a mechanism in which TRIM14 engages distinct deubiquitinase partners to sustain PD-L1 expression. Moreover, the TRIM14–BRCC3 axis represents a promising regulator of PD-L1 protein homeostasis in glioma.

## 2 Materials and methods

### 2.1 Bulk transcriptomic analyses

For comparisons between glioma and non-tumor brain tissue, uniformly processed TCGA and GTEx RNA-sequencing data were obtained from the UCSC Xena Toil recompute compendium. Expression values were analyzed on the log_2_(TPM + 0.001) scale. The analysis included 105 GTEx Brain–Cortex specimens and 662 TCGA primary gliomas, comprising 509 lower-grade gliomas and 153 glioblastomas. TRIM14 expression was compared between non-tumor brain and glioma using a two-sided Wilcoxon rank-sum test.

Disease-associated pathway analysis used two independent human glioma cohorts, TCGA/GTEx and GSE147352. GSE147352 was obtained from the NCBI Gene Expression Omnibus and included 103 glioma samples (85 GBM and 18 LGG) and 15 normal brain samples. It was used for independent TRIM14 expression validation, Hallmark pathway analysis, and cross-dataset comparison. The type-I IFN gene set used for **Figure 1B** was obtained from the Glasgow CVR Orthologous Clusters of Interferon-Stimulated Genes database. Filtering for Homo sapiens, up_regulated genes with log_2_FC > 1 and FDR< 0.05 yielded 918 unique human genes. 777 genes were evaluable in both glioma cohorts and 241 were upregulated in both cohorts. Lowly expressed genes were excluded by retaining genes with an approximate TPM of at least 0.1 in at least 20% of samples in the relevant group. Gene-wise differences between glioma and control brain tissue were estimated using limma, and genes were ranked by the moderated *t* statistic from the glioma-versus-control contrast. Normalized enrichment scores and Benjamini–Hochberg-adjusted FDR values were calculated across the 50 Hallmark gene sets, with positive normalized enrichment scores indicating enrichment toward glioma.

**Figure 1:**
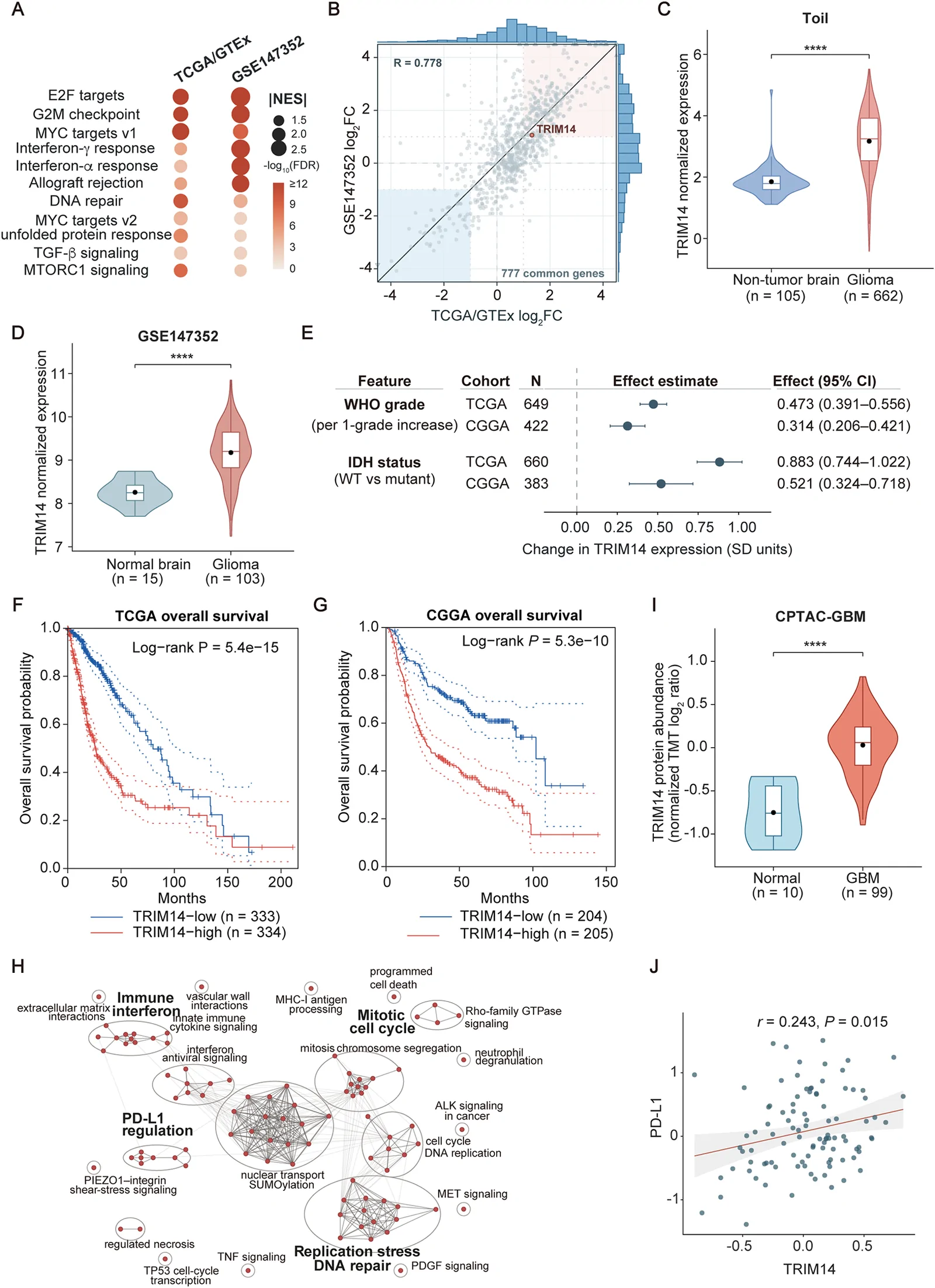
Bulk clinical and proteomic context of TRIM14 in glioma. **(A)** Hallmark pathway landscape in TCGA/GTEx and GSE147352. **(B)** Cross-dataset landscape of type-I interferon genes derived from the Glasgow CVR Orthologous Clusters of Interferon-Stimulated Genes database. **(C)** TRIM14 expression in TCGA/GTEx. **(D)** TRIM14 expression in GSE147352. **(E)** Effect-size forest plot of TRIM14 associations with clinicomolecular features. **(F)** Kaplan–Meier survival curve in TCGA pan-glioma. **(G)** Kaplan–Meier validation in CGGA. **(H)** Network of 95 aggregated bulk Reactome pathways. **(I)** TRIM14 protein abundance in 99 GBM tumors and 10 unmatched GTEx-labeled normal-brain samples assayed within CPTAC/PDC000204. **(J)** Pearson correlation between TRIM14 and PD-L1 protein abundance across 99 GBM tumors. ****P < 0.0001.

For overall survival analysis, TCGA pan-glioma patients were divided using the prespecified TRIM14 median cutoff of 9.2818 (low group, *n* = 331; high group, *n* = 333), and the association was independently evaluated in the CGGA cohort. Overall survival was estimated using the Kaplan–Meier method, and survival distributions were compared using a two-sided log-rank (Mantel–Cox) test.

For analysis of TRIM14 in relation to clinicomolecular features, gene expression and clinical annotations were obtained from the TCGA glioma cohort through UCSC Xena, and an independent expression and clinical dataset was obtained from CGGA for validation of clinicomolecular effect sizes. WHO grade was modeled as a one-grade increase, whereas IDH status was analyzed as wild type versus mutant. TRIM14 expression was standardized within each cohort by one standard deviation (1 SD) to permit comparison of effect estimates across expression scales. Each endpoint was analyzed using samples with complete corresponding annotations.

For bulk Reactome analysis associated with TRIM14, genes were ranked by the moderated *t* statistic for the standardized TRIM14 coefficient in limma–voom models fitted in TCGA and CGGA. Preranked enrichment analysis used the MSigDB 2026.1.Hs Reactome gene sets and fgseaMultilevel, with Benjamini–Hochberg correction within each complete evaluable Reactome universe. TRIM14 was removed from the ranking and gene sets before enrichment analysis. Pathways showing concordant direction and FDR< 0.05 across the two cohorts were organized according to pathway overlap. For visualization, the resulting network was summarized into four manually assigned thematic neighborhoods based on network topology and representative pathways, including immune interferon, mitotic cell cycle, PD-L1 regulation, and replication stress/DNA repair.

### 2.2 Proteomic analyses

Proteomic analyses used the official clean gene-level proteome matrix from CPTAC/PDC study PDC000204. The matrix contained 99 GBM tumor samples and 10 GTEx-labeled normal-brain samples assayed within the same CPTAC GBM proteomics study. These normal samples were not derived from an external GTEx proteomics matrix. **Figure 1I** compared TRIM14 protein abundance between the unmatched GBM and normal-brain groups using an unpaired two-sided Student two-sample *t* test with equal variances assumed. For **Figure 1J**, Pearson correlation between TRIM14 and PD-L1 protein abundance was calculated across 99 GBM tumors. **Supplementary Figure S1G** summarized Pearson correlations between TRIM14 and four prespecified checkpoint proteins (PD-L1/CD274, B7-H3/CD276, CD155/PVR, and HVEM/TNFRSF14), with 95% confidence intervals and Benjamini–Hochberg correction across the four tests. Pairwise complete observations were used; no imputation was applied to missing TNFRSF14 values.

### 2.3 Single-nucleus RNA-sequencing analysis

Single-nucleus RNA-sequencing data from the CARE study were obtained from the NCBI Gene Expression Omnibus under accession GSE326221. Processed cell type and malignant state annotations were obtained from the accompanying CARE Synapse repository, and clinical annotations were obtained from the supplementary tables of the source study. Filtered count matrices and source-study annotations were used for downstream analyses. The paired astrocytoma subset used for the current analyses included 22 patients, 44 specimens (22 Initial and 22 Recurrence specimens), and 206,968 nuclei.

Nuclei with a raw TRIM14 count greater than zero were classified as TRIM14 positive. For **Figure 2A**, the TRIM14 detection fraction was calculated for each sample and source-study major cell type as the number of TRIM14-positive nuclei divided by the total number of nuclei, retaining sample-by-cell-type combinations with at least 20 nuclei. This analysis was descriptive and no formal statistical test was applied.

For visualization, the frozen Full-44 paired astrocytoma atlas comprising 22 patients, 44 samples, and 206,968 nuclei was used. Raw counts were LogNormalized in Seurat with a scale factor of 10,000; 5,000 variable features were selected using the vst method, scaled without covariate regression, and used for principal-component analysis. Harmony integration used PCs 1–30 with case_barcode and laboratory as grouping variables. UMAP coordinates were generated from Harmony dimensions 1–20 using cosine distance, n.neighbors = 40, min.dist = 0.2, and seed = 42. Major cell-type and malignant-state annotations were retained from the source study, and TRIM14 FeaturePlot values were taken from the LogNormalized RNA data layer.

For the malignant-versus-non-malignant glial comparison (**Figure 2D**), raw counts were aggregated at the sample-by-compartment level, with non-malignant glial cells defined as Astrocyte plus Oligodendrocyte. Combinations with at least 20 nuclei were retained, yielding 44 malignant and 39 non-malignant glial profiles. TRIM14 abundance was expressed as log_2_(raw TRIM14 counts/group library size × 10^6^ + 1) and analyzed using a timepoint-adjusted REML linear mixed model with nested patient/sample random intercepts.

**Figure 2:**
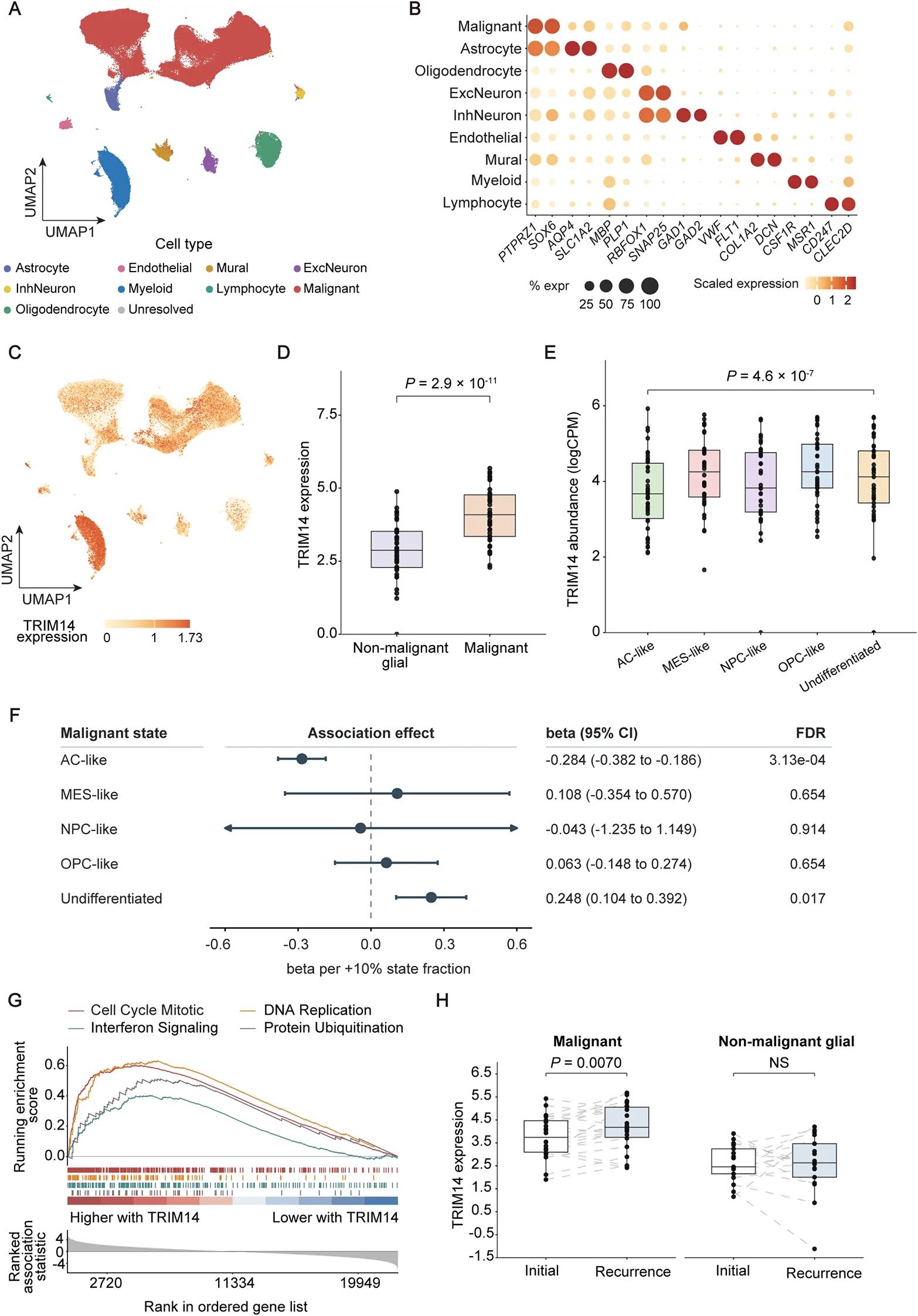
Single-nucleus mapping of TRIM14 across glioma cell populations and longitudinal disease evolution. **(A)** Full-cell atlas of 44 specimens from 22 patients. **(B)** Canonical-marker dot plot used for major cell-type annotation. **(C)** TRIM14 FeaturePlot across all nuclei. **(D)** TRIM14 pseudobulk abundance in malignant and non-malignant glial cells. **(E)** TRIM14 abundance across the AC-like, MES-like, NPC-like, OPC-like, and Undifferentiated malignant states. **(F)** Forest plot of the association between malignant-state architecture and continuous TRIM14, displayed per 10-percentage-point increase in state fraction. **(G)** Overlay of four Reactome pathways associated with continuous TRIM14 in malignant cells. **(H)** Paired Initial–Recurrence comparison of TRIM14 expression in malignant cells (22 pairs) and non-malignant glial cells (17 pairs).

For malignant-state analysis, raw counts were aggregated for sample-by-state combinations containing at least 20 malignant nuclei across AC-like, MES-like, NPC-like, OPC-like, and Undifferentiated states. TRIM14 abundance was expressed as log2(state-specific CPM + 1) and analyzed using a REML linear mixed model with malignant state and timepoint as fixed effects and nested patient/sample random intercepts. The omnibus state test was followed by all 10 pairwise contrasts with Benjamini–Hochberg-adjusted *P* values. For state-architecture analysis, state fractions were defined within each sample as the number of nuclei in a given malignant state divided by all malignant nuclei. Associations between standardized TRIM14 abundance and state fraction were estimated with timepoint adjustment using patient-clustered CR2 standard errors and Satterthwaite degrees of freedom, and displayed per 10-percentage-point increase in state fraction.

For whole-malignant continuous-TRIM14 transcriptional association analysis (**Figure 2G**), all-gene raw-count pseudobulk profiles were filtered with filterByExpr, TMM-normalized, and analyzed with limma–voom using standardized TRIM14 and timepoint as predictors. Repeated patients were accounted for using duplicateCorrelation. Genes were ranked by the moderated *t* statistic for the TRIM14 coefficient. Reactome enrichment used MSigDB v2026.1.Hs C2:CP: REACTOME gene sets and fgseaMultilevel (minSize = 15, maxSize = 500), with TRIM14 removed from both the ranking and gene sets and Benjamini–Hochberg correction across all eligible Reactome sets.

For the myeloid supplementary analyses, Microglia-like cells were defined as source-study clusters c1+c7 and Macrophage-like cells as c3+c5. Sample-by-lineage raw-count pseudobulk profiles with at least 10 nuclei were TMM-normalized. The cross-lineage comparison in **Supplementary Figure S2B** used a patient-aware, timepoint-adjusted mixed-model framework. Longitudinal lineage-specific analyses in **Supplementary Figure S2C** used TRIM14 log_2_CPM as the outcome with timepoint as a fixed effect and patient as a random intercept. Benjamini–Hochberg correction was applied across the two lineage-specific longitudinal tests. **Supplementary Figure S2D** summarized eight compartment-specific TRIM14-associated Reactome pathways across malignant, microglia-like, and macrophage-like compartments as a qualitative matrix. No formal compartment-by-TRIM14 interaction test was performed.

Longitudinal malignant-cell analysis used all-gene malignant pseudobulk profiles from 22 complete Initial–Recurrence pairs (44 samples), with TMM normalization and a two-sided paired Wilcoxon signed-rank test. Non-malignant glial analysis combined Astrocyte and Oligodendrocyte, used filterByExpr followed by TMM normalization with prior.count = 2, and included 17 complete Initial–Recurrence pairs (34 samples). The two compartments were normalized independently and analyzed separately. No cross-compartment interaction test was performed.

### 2.4 Animal treatment

Six-week-old male C57BL/6J mice were purchased from Guangdong Provincial Experimental Animal Center. All mice were housed in a specific-pathogen-free (SPF) barrier environment at Sun Yat-sen University. Experimental procedures were performed with the approval of Animal Care and Use Committee. 1 × 10^6^ GL261 cells in 100 μL of PBS were subcutaneously inoculated into the flank of mice to establish a subcutaneous tumor model. Tumor growth was monitored every two days, and tumor volume was calculated using the formula as previously described (9):


$$Tumor\;Volume=\frac{\pi }{6}\times length\times width\times thickness$$

### 2.5 Cell culture

LN229 and GL261 were obtained from the Sun Yat-sen University Cancer Center. Cells were maintained in complete Dulbecco’s Modified Eagle Medium (Gibco) supplemented with 10% fetal bovine serum and 1% L-glutamine (Gibco) in a humidified incubator with 37 °C and 5% CO2. Cells were routinely passaged upon reaching 80–90% confluence.

### 2.6 Western blot analysis

Cells were lysed on ice for 30 min in lysis buffer supplemented with protease inhibitors. After centrifugation at 4 °C, the supernatants were collected. Proteins were separated by SDS-PAGE and then transferred onto PVDF membranes, which were blocked with 5% non-fat milk dissolved in TBST (Tris-buffered saline (pH 7.4) containing 0.1% Tween-20) and incubated with corresponding primary antibodies overnight at 4 °C. On the following day, the membranes were washed three times with TBST, incubated with secondary antibodies, washed again, and finally visualized using chemiluminescence detection.

### 2.7 Co-immunoprecipitation

For Co-IP assays, cells were lysed on ice for 30 min using RIPA lysis buffer. Lysates were centrifuged for 10 min, and the collected supernatants were incubated with antibody-conjugated beads at 4 °C with constant rotation overnight. After washing five times with wash buffer, beads were mixed with SDS-PAGE loading buffer and heated at 95 °C for 10 min to elute bound proteins. The co-immunoprecipitated proteins were subsequently analyzed by western blot as described above.

### 2.8 RNA extraction and quantitative real-time PCR

Total RNA was extracted using an EZB RNA extraction kit. After removal of genomic DNA, the RNA was reverse transcribed into cDNA using the RT-qPCR Fast Master Mix. Quantitative real-time PCR was performed with 2× PolarSignal SYBR Green Mix Taq (with Tli RNaseH; MIKX), and the threshold cycle (Ct) values for each target gene were normalized to endogenous reference genes (human β-actin or mouse Gapdh) to calculate relative expression levels using the 2−ΔΔCt method. The paired primers used in this study were as follows:

Human *β-actin* forward: 5’-CATGTACGTTGCTATCCAGGC-3’

Human *β-actin* reverse: 5’-CTCCTTAATGTCACGCACGAT-3’

Human *CD274* forward: 5’-TGGCATTTGCTGAACGCATTT-3’

Human *CD274* reverse: 5’-TGCAGCCAGGTCTAATTGTTTT-3’

### 2.9 Plasmids and siRNA transfection

For plasmid transfection, DNA and Lipofectamine 3000 were separately diluted in Opti-MEM medium at a ratio of 1 μg DNA to 1 μL Lipofectamine 3000. For siRNA transfection, Lipofectamine RNAi MAX (Invitrogen) was used according to the manufacturer’s instructions. The diluted mixtures were thoroughly vortexed, briefly centrifuged, and incubated at room temperature for 10 min prior to addition to cell cultures. The Myc/HA-TRIM14, Myc-BRCC3 and Myc-USP14 expression constructs were obtained from School of Life Science, Sun Yat-sen University (SYSU). HA-tagged ubiquitin variants, including ubiquitin-WT, ubiquitin-K6, -K11, -K27, -K33, -K48, and -K63 were also obtained from School of Life Science, Sun Yat-sen University (SYSU). TRIM14 full-length and truncation constructs (ΔABB, ΔABC, ΔPRY, PRY) were generated by standard PCR-based cloning and verified by DNA sequencing. Knockdown efficiency was validated by quantitative real-time PCR and immunoblot analysis at 48 h post-transfection. All experiments were performed with at least three independent biological replicates. The siRNA sequences used in this study were as follows:

human *TRIM14*-siRNA #1 forward: CAGAUUACUACUUGACGAA

reverse: UUCGUCAAGUAGUAAUCUG

human *TRIM14*-siRNA #2 forward: CCAAGAAAUUCAUUGAUAA

reverse: UUAUCAAUGAAUUUCUUGG

human *BRCC3*-siRNA #1 forward: GCAGGAAUUACAACAAGAA

reverse: UUCUUGUUGUAAUUCCUGC

human *BRCC3*-siRNA #2 forward: GAAGGACCGAGUAGAAAUU

reverse: AAUUUCUACUCGGUCCUUC

### 2.10 Construction of knockout (KO) cell lines

The sgRNAs targeting *TRIM14*, *ATG5* and *SQSTM1* were synthesized by Sangon Biotech (Shanghai, China) and inserted into LentiCRISPRv2 vector followed by virus packaging and purification. The purified virus was then used to infect the pre-plated target cells, and stably transfected cells were obtained by screening with the corresponding antibiotic for a certain period. After isolating single-cell clones by the limiting dilution method, the knockout efficiency of positive clones was identified by detecting the mRNA level of the target gene via qPCR and the protein expression level through Western blotting.

### 2.11 Reagents and antibodies

The following reagents were used at the indicated concentrations and durations: cycloheximide (CHX, 50 μg/mL, 3 h, 6 h), MG132 (10 μM, 4 h), 3-methyladenine (3-MA, 5 mM, 6 h), bafilomycin A1 (BafA1, 200 nM, 6 h), Torin1 (250 nM, 6 h, 9 h), and EBSS starvation medium (6 h, 9 h). All reagents were dissolved in DMSO or appropriate solvents as per the manufacturer’s instructions, and equivalent volumes of the corresponding solvents were used as vehicle controls in all experiments.

The following primary antibodies were used for immunoblotting (WB), immunoprecipitation (IP), and immunofluorescence (IF): anti-PD-L1 (WB 1:2000, IP 1 μg, IF: 1:200), anti-TRIM14 (WB 1:1000), anti-BRCC3 (WB 1:1000), anti-USP14 (WB 1:1000), anti-p62 (WB 1:1000), anti-LC3B (WB 1:1000), anti-Flag (WB 1:3000), anti-HA (WB 1:2000, IF: 1:2000), anti-K63-linked ubiquitin (WB 1:1000), anti-total ubiquitin (WB 1:1000), and anti-β-actin (WB 1:3000), DAPI (IF: 1:10000). Horseradish peroxidase (HRP)-conjugated secondary antibodies (anti-rabbit and anti-mouse) were used at 1:2000 dilution for immunoblotting; Alexa Fluor-conjugated secondary antibodies (488, 568, 647) were used at 1:1000 dilution for immunofluorescence. All antibody dilutions were prepared in blocking buffer (5% non-fat milk in TBST for WB, or 3% BSA in PBS for IF) and validated in each experimental system prior to use.

### 2.12 Flow cytometry

Tumor sections were digested in collagenase IV (Worthington, 3 mg/mL) and DNase I (Sigma-Aldrich, 0.02%) at 37 °C for 1 hour, followed by filtration through cell strainers. Single-cell suspensions were washed in PBS. Then, cells were stained with anti-CD3-APC (BioLegend), anti-CD4-PE (BioLegend), and anti-CD8-FITC (BioLegend). After washing with PBS, cell suspensions were subjected to flow cytometry (Beckman CytoFLEX) for analysis. Statistical analysis was performed by Flowjo_v10.

### 2.13 Statistical analysis

The data calculation was performed as described above. Other quantification was performed by Prism 8 (GraphPad Software). Statistical significance was determined using two-tailed unpaired t-tests or two-way ANOVA, with *P* < 0.05 considered significant.

## 3 Results

### 3.1 TRIM14 was associated with glioma progression

We first evaluated disease-associated transcriptional programs in two independent human glioma cohorts. IFN-alpha and IFN-gamma response signatures were significantly enriched in both TCGA/GTEx and GSE147352 (**Figure 1A**; NES 1.66 and 1.61 in TCGA/GTEx, and 2.69 and 2.77 in GSE147352, respectively), indicating a consistent association between interferon-related immune programs and the glioma state. Among the 777 type-I IFN-related genes detected in both cohorts, log_2_ fold-changes were highly concordant (Pearson R = 0.778). 241 genes were upregulated in both cohorts, and TRIM14 showed the same tumor-associated direction of change (**Figure 1B**). In independent expression comparisons, the TCGA/GTEx analysis included 662 gliomas and 105 GTEx Brain–Cortex specimens, whereas GSE147352 included 103 gliomas and 15 normal brain specimens. In GSE147352, TRIM14 showed a log_2_FC of 1.06 with an FDR of 1.77 × 10^-9^ (**Figures 1C, D**).

TRIM14 expression was concordant with clinicomolecular features and outcome. TRIM14 increased with WHO grade (TCGA: beta = 0.473 per grade, 95% CI 0.391–0.556, *n* = 649; CGGA: beta = 0.314, 95% CI 0.206–0.421, *n* = 422) and was higher in IDH-wildtype than in IDH-mutant tumors (TCGA: beta = 0.883, 95% CI 0.744–1.022, *n* = 660; CGGA: beta = 0.521, 95% CI 0.324–0.718, *n* = 383) (**Figure 1E**; **Supplementary Figures S1A–F**). In TCGA pan-glioma, higher TRIM14 expression was associated with shorter overall survival using the prespecified median cutoff (9.2818; low, *n* = 331; high, *n* = 333), with independent validation in CGGA (**Figures 1F, G**). The TRIM14-associated bulk Reactome network was organized around four major thematic neighborhoods: immune interferon, mitotic cell cycle, PD-L1 regulation, and replication stress/DNA repair (**Figure 1H**). In the CPTAC/PDC000204 proteome, TRIM14 protein abundance was higher in 99 GBM tumors than in 10 unmatched GTEx-labeled normal-brain samples assayed within the same proteomics study (two-sided equal-variance Student t test, *t* = 6.77, *P* = 7.23 × 10^-10^). Across the 99 GBM tumors, TRIM14 protein abundance was positively correlated with PD-L1 protein abundance (Pearson *r* = 0.243, 95% CI 0.048–0.421, *P* = 0.015; **Figures 1I, J**; **Supplementary Figure S1G**).

To resolve the cellular distribution of TRIM14 in the glioma ecosystem, we analyzed paired CARE single-nucleus RNA-sequencing data from 22 patients and 44 specimens comprising 206,968 nuclei. TRIM14 RNA was detected across major cell populations (**Figures 2A–C**; **Supplementary Figure S2A**), and malignant cells showed higher pseudobulk TRIM14 abundance than non-malignant glial cells (difference = +1.1865 log_2_(CPM + 1), 95% CI 0.8817–1.4914, *P* = 2.9 × 10^-11; **Figure 2D**). Within malignant cells, TRIM14 was detected across the AC-like, OPC-like, NPC-like, MES-like, and Undifferentiated states, with an overall difference in abundance across states (F(4,146) = 9.82, *P* = 4.57 × 10^-7^; **Figure 2E**). State architecture was associated with continuous TRIM14 abundance: the AC-like fraction showed a negative association (beta = −0.284, FDR = 3.13 × 10^-4^), whereas the Undifferentiated fraction showed a positive association (beta = 0.248, FDR = 0.0167) (**Figure 2F**). Whole-malignant TRIM14-associated gene rankings showed enrichment of cell cycle/mitotic, DNA replication, interferon signaling, and protein ubiquitination Reactome programs (**Figure 2G**). Longitudinally, malignant-cell TRIM14 expression increased from Initial to Recurrence across 22 complete pairs (paired Wilcoxon *P* = 0.0070), whereas no significant change was observed in non-malignant glial cells across 17 complete pairs (*P* = 0.813; **Figure 2H**). Related myeloid-compartment results are presented in **Supplementary Figure S2**.

### 3.2 TRIM14 stabilized PD-L1 in glioma cells

We next sought to further determine the positive association between TRIM14 and PD-L1. Knockdown of TRIM14 reduced PD-L1 protein abundance, whereas ectopic TRIM14 increased PD-L1 protein levels in glioma cells (**Figures 3A–C**). In contrast, *CD274* mRNA was not materially changed by either TRIM14 overexpression or knockdown (**Figures 3D, E**). These findings indicate that TRIM14 regulates PD-L1 at the protein level.

**Figure 3:**
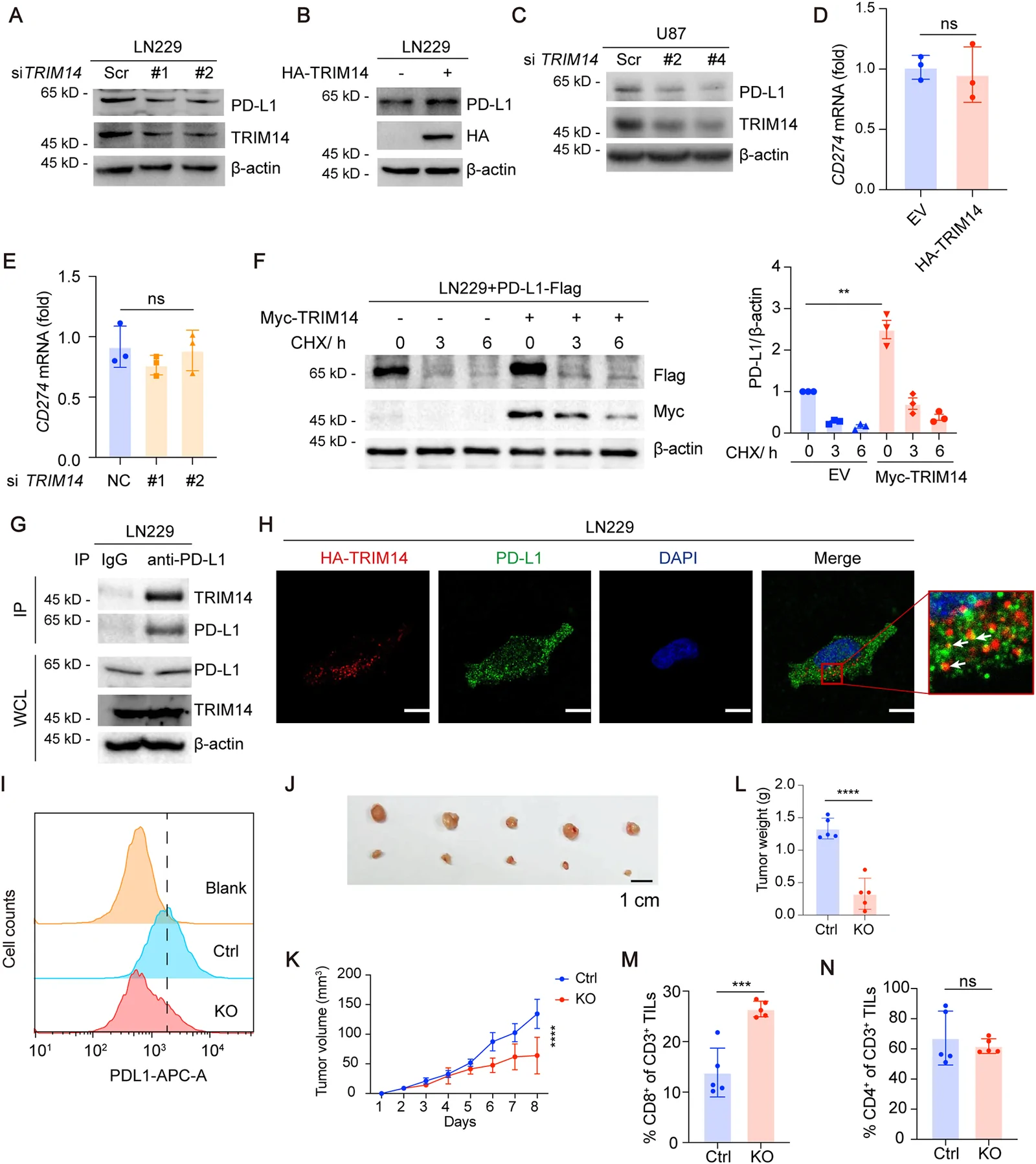
TRIM14 binds to and stabilizes PD-L1. **(A)** Immunoblot analysis of PD-L1 after TRIM14 knockdown with *TRIM14* siRNAs in LN229 cells. **(B)** PD-L1 abundance after ectopic HA-TRIM14 expression. **(C)** Immunoblot analysis of PD-L1 after TRIM14 knockdown with *TRIM14* siRNAs in U87 cells. **(D, E)** *CD274* mRNA expression levels after TRIM14 overexpression **(D)** or knockdown **(E)**. Data are mean ± SD with *P* values an unpaired two-tailed Student *t* test. **(F)** Cycloheximide-chase (CHX) analysis of PD-L1 in cells expressing empty vector (EV) or TRIM14, with densitometric quantification. Data are mean ± SD with P values an unpaired two-tailed Student *t* test. **(G)** Endogenous Co-immunoprecitation (Co-IP) of TRIM14 with PD-L1. **(H)** Confocal imaging of HA-TRIM14 (red), PD-L1 (green), and DAPI (blue). Arrows indicate colocalized puncta. Scale bar, 100 μm. **(I)** Control (Ctrl) and *Trim14*-KO GL261 cells were stained with anti-PD-L1-APC antibody and analyzed by flow cytometry analysis. **(J)** Representative tumors generated from control and *Trim14*-knockout GL261 cells. **(K)** Tumor volume curves. Data are mean ± SD with a two-way ANOVA test (*n* = 5 mice per group). **(L)** Endpoint tumor weights. Data are mean ± SD with *P* values from an unpaired two-tailed Student *t* test. **(M, N)** Flow cytometry analysis of the CD8^+^ populations **(M)** or CD4^+^ populations **(N)** in CD3^+^ T cells from GL261 tumors. The data are shown as the mean ± SD, with *P* values based on an unpaired two-tailed Student t test (*n* = 5). ns, not significant; **P < 0.01; ***P < 0.001; ****P < 0.0001.

With cycloheximide (CHX) chase experiments, PD-L1 declined rapidly in control cells but remained more abundant in TRIM14 overexpressing cells (**Figure 3F**). Endogenous co-immunoprecipitation (Co-IP) demonstrated an association between TRIM14 and PD-L1 (**Figure 3G**). Confocal imaging further showed colocalization of TRIM14 and PD-L1 signals (**Figure 3H**).

Flow cytometry assays showed that *Trim14* knockout decreased cell-surface PD-L1 levels (**Figure 3I**), which is considered as regulating exhaustion of T cells. Therefore, we examined the role of TRIM14 in tumor progression in a subcutaneous GL261 tumor model. *Trim14* deletion suppressed tumor growth in C57BL/6J mice bearing subcutaneous GL261 tumors, and reduced tumor weight (**Figures 3J–L**). Additionally, *Trim14* knockout significantly increased infiltration of CD8^+^ T cells but not CD4^+^ T cells in tumor sections (**Figures 3M, N**). These data support that TRIM14 stabilized PD-L1, and promoted glioma growth.

### 3.3 TRIM14 suppressed p62-mediated selective autophagic degradation of PD-L1

We next determined the degradation pathway opposed by TRIM14. Proteasome inhibition with MG132 did not eliminate the difference in PD-L1 abundance between control and TRIM14-expressing cells. By contrast, 3-MA and BafA1 largely occluded the PD-L1-stabilizing effect of TRIM14 (**Figure 4A**), implicating autophagy–lysosome-dependent turnover. Consistently, PD-L1 decreased during EBSS starvation or Torin1 treatment, whereas TRIM14 expression attenuated this loss (**Figures 4B, C**). In *ATG5*-knockout cells, PD-L1 was stabilized and TRIM14 no longer produced a clear additional increase (**Figure 4D**). These genetic and pharmacologic results place TRIM14 upstream of canonical autophagic PD-L1 clearance.

**Figure 4:**
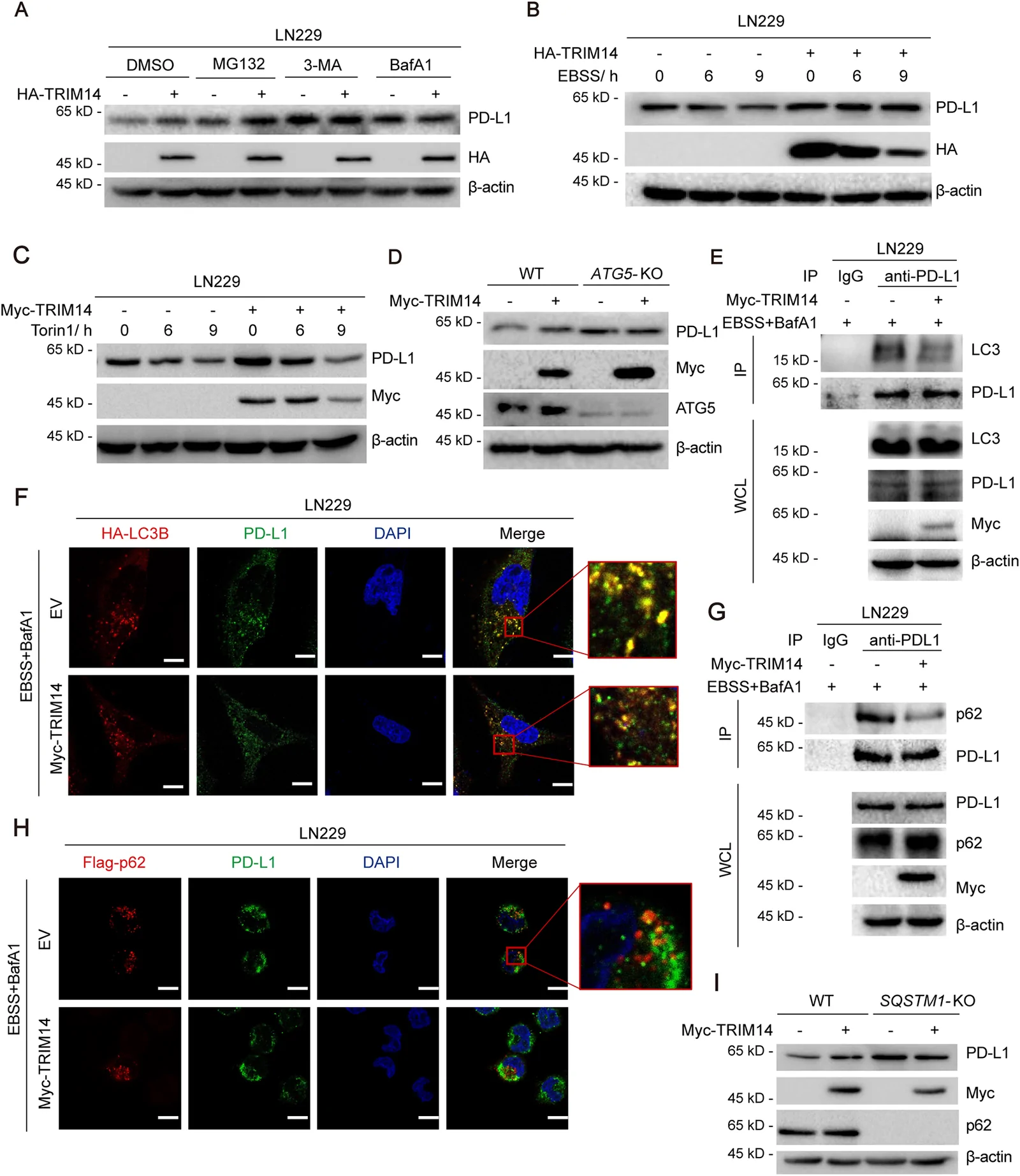
TRIM14 inhibits p62-dependent selective-autophagic degradation of PD-L1. **(A)** PD-L1 abundance in LN229 cells expressing empty vector or HA-TRIM14 after treatment with DMSO, MG132, 3-MA, or bafilomycin A1 (BafA1). **(B, C)** PD-L1 during EBSS starvation **(B)** or Torin1 treatment **(C)** in the presence or absence of ectopic TRIM14. **(D)** PD-L1 in wild-type (WT) and *ATG5*-knockout cells with or without expression of TRIM14. **(E)** Co-IP analysis of LC3 and PD-L1 under indicated conditions. **(F)** Confocal imaging of HA-LC3B (red), PD-L1 (green), and DAPI (blue) in cells expressing empty vector or TRIM14. Magnified regions show colocalization of PD-L1 and LC3. Scale bar, 100 μm. **(G)** Co-IP analysis of p62 with PD-L1 with or without ectopic TRIM14. **(H)** Confocal imaging of Flag-P62 (red), PD-L1 (green), and DAPI (blue). **(I)** PD-L1 abundance in WT and *SQSTM1*-knockout cells expressing TRIM14. Scale bar, 100 μm.

Selective autophagy requires recruitment of ubiquitinated cargo to LC3-positive autophagosomes through cargo receptors. Co-immunoprecipitation and imaging showed that TRIM14 reduced PD-L1 association and colocalization with LC3 under autophagy-inducing conditions (**Figures 4E, F**). TRIM14 also reduced the association between PD-L1 and p62 (**Figure 4G**), whereas the p62 imaging pattern did not show a comparable TRIM14-dependent redistribution (**Figure 4H**). In *SQSTM1*-knockout cells, PD-L1 accumulated and TRIM14 failed to confer further stabilization (**Figure 4I**). These observations identify p62 as the functionally relevant cargo receptor and support a model in which TRIM14 prevents delivery of PD-L1 to the autophagic machinery.

### 3.4 BRCC3 was required for K63-linked deubiquitination and stabilization of PD-L1

p62 recognized ubiquitin chains as cargo-recognition signals (15). Therefore, we wondered whether TRIM14 altered PD-L1 ubiquitination. TRIM14 expression decreased total ubiquitin chains of immunoprecipitated PD-L1, and specifically reduced K63-linked ubiquitination (**Figures 5A–C**). Additionally, analysis of single-linkage ubiquitin variants supported K63 as the dominant linkage affected by TRIM14 (**Figure 5B**).

**Figure 5:**
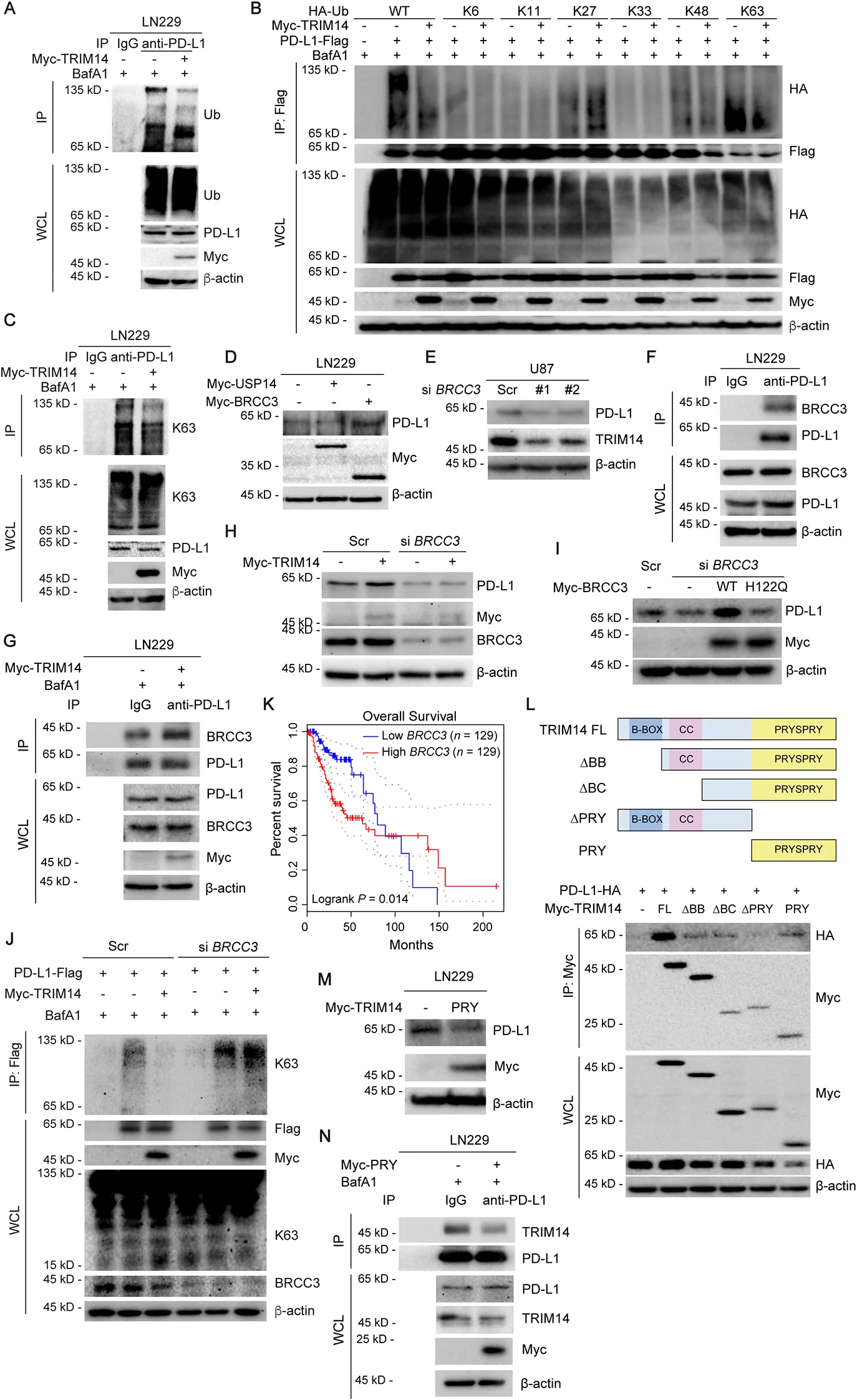
BRCC3 is required for K63 deubiquitination and stabilization of PD-L1. **(A)** Ubiquitination of immunoprecipitated PD-L1 in LN229 cells expressing TRIM14 or not with treatment of BafA1. **(B)** Ubiquitination of PD-L1 in cells co-transfected with PD-L1-Flag and distinct HA-tagged ubiquitin variants (WT, K6, K11, K27, K33, K48, K63), with indicated treatment. **(C)** K63-linked ubiquitination of endogenous PD-L1 in LN229 with overexpression of TRIM14 or not after BafA1 treatment. **(D)** PD-L1 abundance after ectopic USP14 or BRCC3 expression. **(E)** Immunoblot analysis of PD-L1 after BRCC3 knockdown with *BRCC3* siRNAs in U87 cells. **(F)** Co-IP analysis of BRCC3 and PD-L1. **(G)** Co-IP analysis of BRCC3 and PD-L1 with TRIM14 overexpression. **(H)** Immunoblot analysis of PD-L1 levels in control (Scr) or *BRCC3* knockdown U87 cells transfected with Myc-TRIM14. **(I)** PD-L1 levels in control (Scr) or *BRCC3* knockdown U87 cells transfected with empty vector, wild type BRCC3 (WT) or BRCC3 mutant (H122Q). **(J)** Co-IP and immunoblot analysis of K63-linked ubiquitination of PD-L1 in control (Scr) or *BRCC3* knockdown HEK293T cells transfected with PD-L1-Flag or Myc-TRIM14, followed by BafA1 treatment. **(K)** Overall survival of patients with low or high BRCC3 expression. **(L)** Co-IP analysis of PD-L1 and full length TRIM14 or domain deficient constructs. **(M)** PD-L1 abundance after expression of TRIM14 PRYSPRY region. **(N)** Co-IP analysis of TRIM14 and PD-L1 with or without ectopic TRIM14 PRYSPRY domain.

TRIM14 lacks intrinsic deubiquitinase activity and therefore was expected to recruit a catalytic partner. Previously, we found TRIM14 recruited deubiquitinases USP14 and BRCC3 to remove K63-linked ubiquitination of substrate proteins (12, 16). Here we found BRCC3 increased PD-L1 protein levels, whereas USP14 showed little or no comparable effect in LN229 cells (**Figures 5D, E**). Endogenous Co-IP analysis confirmed association of BRCC3 with PD-L1 (**Figure 5F**), and TRIM14 overexpression promoted this association (**Figure 5G**). TRIM14 failed to stabilize PD-L1 in BRCC3-knockdown cells (**Figure 5H**), indicating that TRIM14 relied on BRCC3 to stabilize PD-L1. To investigate the role of deubiquitinase activity, we constructed a plasmid expressing BRCC3 mutant (H122Q) which is deficient in deubiquitinase activity. Ectopic expression of wild-type BRCC3 increased PD-L1 levels in BRCC3 knockdown cells, while BRCC3 mutant failed to restore PD-L1 levels (**Figure 5I**). Consistently, overexpression of TRIM14 failed to reduce K63-linked ubiquitination of PD-L1 in BRCC3-knockdown cells (**Figure 5J**). These results supported the conclusion that BRCC3 is indispensable for TRIM14-mediated PD-L1 stabilization and deubiquitination. High BRCC3 expression was also associated with worse overall survival of glioma patients (**Figure 5K**).

We next investigated interacting domains between TRIM14 and PD-L1. Domain deletion assays revealed that full-length TRIM14 bound PD-L1 most efficiently, and that the interaction was primarily mediated through the PRYSPRY region of TRIM14 (**Figure 5L**). However, the isolated PRYSPRY region was insufficient for PD-L1 stabilization. Instead, it led to a reduction in PD-L1 protein levels (**Figure 5M**). We overexpressed TRIM14 PRYSPRY domain to investigate its role in interaction between TRIM14 and PD-L1. Co-immunoprecipitation analysis showed that ectopic TRIM14 PRYSPRY domain decreased the interaction between TRIM14 and PD-L1 (**Figure 5N**), indicating that TRIM14 PRYSPRY domain competitively interacted with PD-L1 to interfere with endogenous TRIM14-mediated stabilization. However, its potential as a dominant-negative inhibitor requires further experimental validation.

On the basis of these findings, we proposed that TRIM14 functioned as a scaffold to recruit BRCC3, which removed K63-linked ubiquitin of PD-L1, attenuated p62 recognition, and consequently suppressed selective autophagic degradation of PD-L1 in glioma. Given that the PRYSPRY domain is essential for TRIM14-PD-L1 interaction and TRIM14-mediated PD-L1 stabilization, targeting this interaction interface may offer a strategy for destabilizing PD-L1 in glioma, although this remains to be tested in future studies.

## 4 Discussion

This study identified a pathway that connected an inflammation-associated scaffold protein to immune-checkpoint stability in glioma. In clinical data analysis, TRIM14 was associated with poor outcome, interferon and inflammatory programs, CD274 expression, and disease evolution. Mechanistically, TRIM14 bound PD-L1 and increased its protein stability without altering *CD274* mRNA. Our observations supported a model in which TRIM14 recruited BRCC3 to erase the ubiquitin signal that enabled p62-dependent delivery of PD-L1 to autophagosomes, thereby sustaining checkpoint abundance.

In single-nucleus RNA-sequencing data, TRIM14-positive nuclei were predominantly malignant, with myeloid cells representing the second largest compartment. Within the malignant population, TRIM14 was detected across AC-like, OPC-like, NPC-like, MES-like, and undifferentiated states. The increase in TRIM14 expression in malignant cells from later paired samples was consistent with its induction during glioma progression. Future analyses will be required to determine whether this change is driven by recurrence, treatment exposure, shifts in cellular composition, or other contributing factors.

Our findings also contributed to the emerging understanding of TRIM14 biology. Previous studies have shown that TRIM14 protects cGAS, p100/p52, and KDM4D from selective autophagic degradation and promotes inflammatory signaling (12, 16, 17). TRIM14 was also found to recruit USP14 to deubiquitinate and stabilize PD-L1 in breast cancer models (12). In this work, we revealed a related yet distinct mechanism in glioma, in which BRCC3, rather than USP14, is the required deubiquitinase. This difference is biologically plausible, as TRIM14 functions as a scaffold but not a catalytic enzyme, and can assemble context-dependent complexes. BRCC3 is a K63-directed JAMM-family deubiquitinase that participates in the regulation of inflammatory proteins and has previously been implicated in glioma survival and temozolomide resistance (18, 19). Accordingly, the effect of the TRIM14-BRCC3 axis on PD-L1 broadens the repertoire of both proteins, extending their known involvement from stress adaptation and inflammatory responses to the maintenance of checkpoint proteostasis.

The mechanistic distinction between transcriptional induction and protein stabilization has therapeutic implications. Interferons and inflammatory signaling can increase *CD274* transcription, while TRIM14–BRCC3 can prevent the degradation of PD-L1 protein. These architectures may allow glioma cells to maintain checkpoint signaling even when upstream transcription fluctuates. Targeting TRIM14–BRCC3 would be expected to decrease PD-L1 through intracellular degradation rather than antibody-mediated extracellular blockade. However, whether deficiency of TRIM14 or BRCC3 affects T-cell immune function or the efficacy of immune-checkpoint blockade remains unknown. Additionally, the *in vivo* experiments in this study were performed using a subcutaneous GL261 graft model, which does not fully recapitulate the intracranial tumor microenvironment, blood-brain barrier, or the unique immune ecosystem of glioma. Therefore, whether the TRIM14-BRCC3-PD-L1 axis exerts similar immunomodulatory functions in the orthotopic brain tumor setting remains to be determined. Future studies using intracranial glioma models will be necessary to validate the translational relevance of our findings. Development of inhibitors must also consider the broader roles of BRCC3 in DNA repair and innate immunity, as systemic inhibition could produce both therapeutic synergy and toxicity.

This study has several limitations. We investigated the role of TRIM14 in glioma progression via a subcutaneous GL261 tumor model which lacks the intracranial microenvironment, blood-brain barrier and immune ecosystem of glioma. What’s more, we did not assess effects of immunotherapy in combination with therapies targeting TRIM14 or PRYSPRY domain, T cell activation, cytotoxic killing or cytokine release. Therefore, it is insufficient to support glioma-specific immunotherapeutic conclusions.

In conclusion, our data support a glioma progressed mechanism where the TRIM14–BRCC3 axis stabilizes PD-L1 by removing K63-linked ubiquitin and preventing p62-dependent selective autophagy. The study also provides a framework for understanding how inflammatory adaptation is coupled to immune checkpoint persistence. Targeting this pathway may offer a route to destabilize PD-L1 and sensitize glioma to immune therapy, pending further validation in clinically relevant models.

## Data Availability

The original contributions presented in the study are included in the article/**Supplementary Material**. Further inquiries can be directed to the corresponding author.

## Supplementary material

Supplementary Figure S1 Clinicomolecular and survival associations of TRIM14. **(A)** TRIM14 expression across TCGA WHO grades. **(B)** TRIM14 expression across CGGA WHO grades. **(C)** TRIM14 expression by IDH status in TCGA. **(D)** TRIM14 expression by IDH status in CGGA. **(E)** Univariable Cox subgroup analysis of continuous TRIM14 per 1-SD increase in all glioma, LGG, and GBM. **(F)** Multivariable Cox analysis including age, WHO grade, IDH status, and 1p/19q codeletion. **(G)** Pearson protein-correlation forest plot for TRIM14 with PD-L1/CD274, B7-H3/CD276, CD155/PVR, and HVEM/TNFRSF14, showing 95% confidence intervals and Benjamini–Hochberg FDR across the four prespecified tests.

Supplementary Figure S2 TRIM14 context in myeloid compartments. **(A)** TRIM14-positive nuclear fraction by sample and major cell type. **(B)** TRIM14 pseudobulk abundance in microglia-like and macrophage-like compartments. **(C)** Longitudinal change in TRIM14 across Initial and Recurrence in the two myeloid compartments. **(D)** Matrix of eight TRIM14-associated Reactome pathways across malignant, microglia-like, and macrophage-like compartments.
