# Supplementary material for: TRIM14 recruits BRCC3 to stabilize PD-L1 by suppressing p62-dependent selective autophagy in glioma
Source: Front Immunol. 2026 Oct 13;17:1942769. doi: 10.3389/fimmu.2026.1942769 (PMC13710066; doi:10.3389/fimmu.2026.1942769)
Supplement: Supplementary file 1 [file DataSheet1.pdf]

F3

A

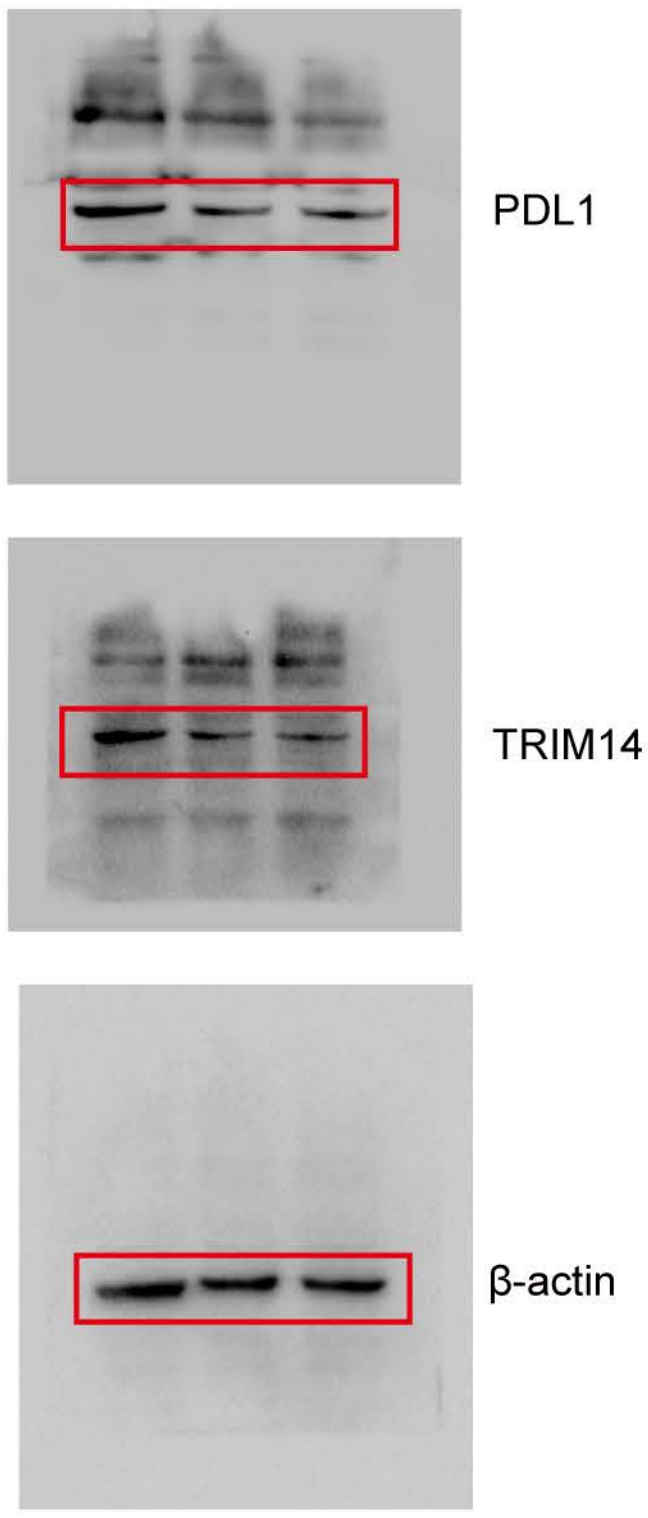
PDL1
TRIM14
β-actin

B

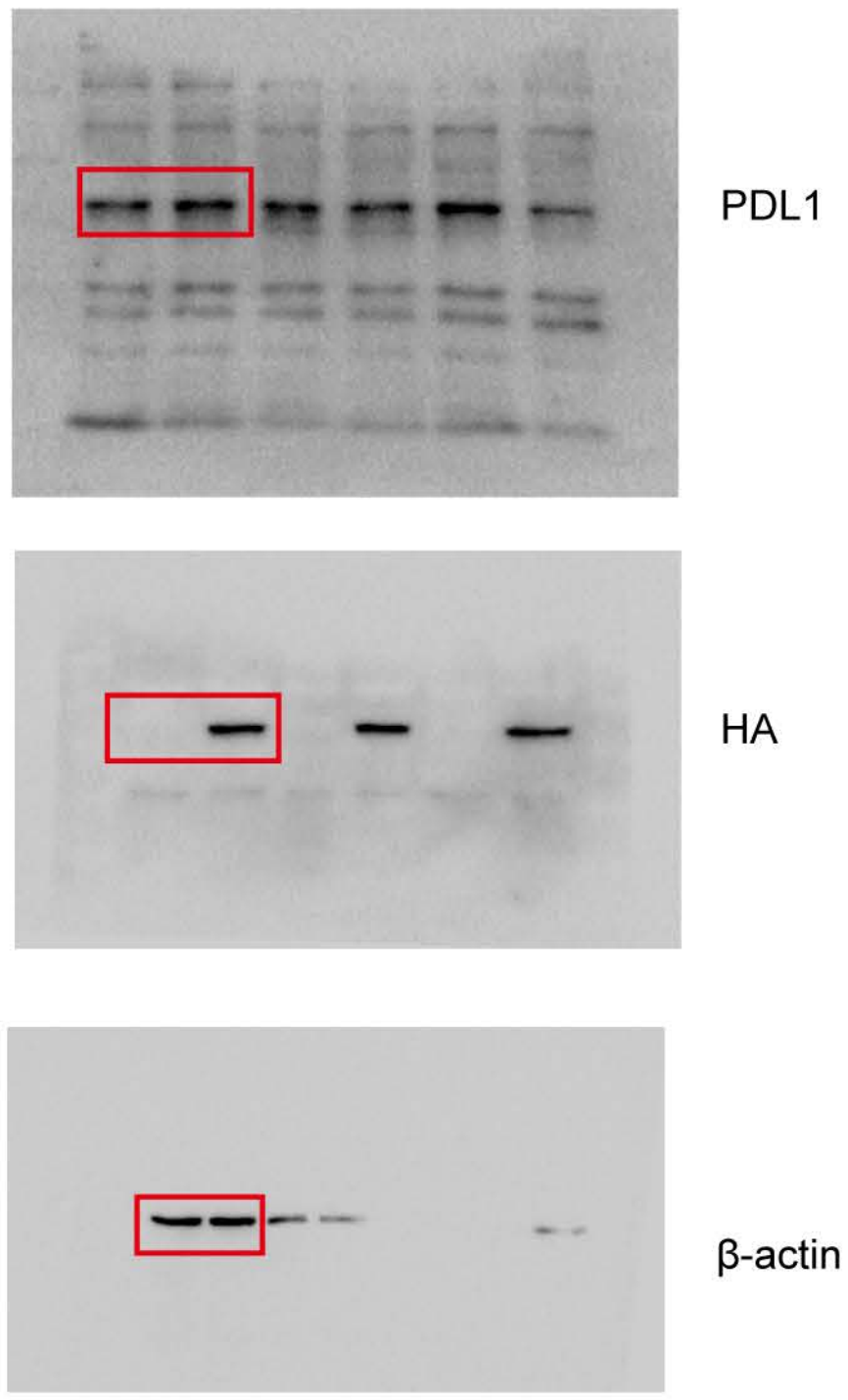
PDL1
HA
β-actin

G

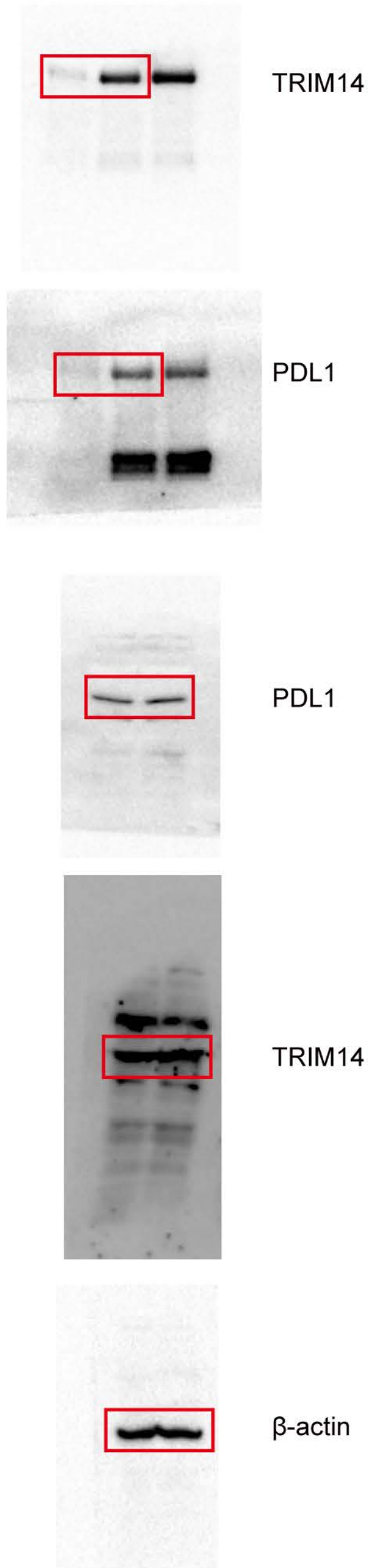
TRIM14
PDL1
PDL1
TRIM14
β-actin

C

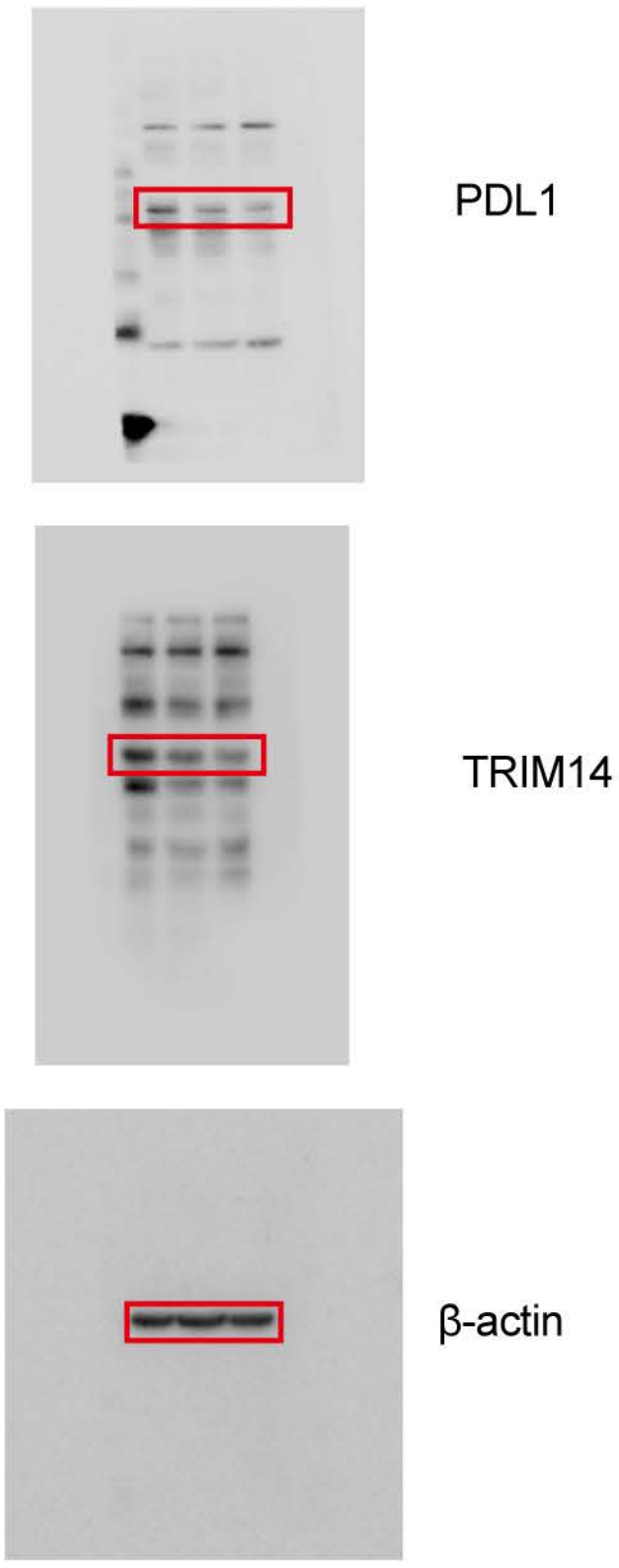
PDL1
TRIM14
β-actin

F

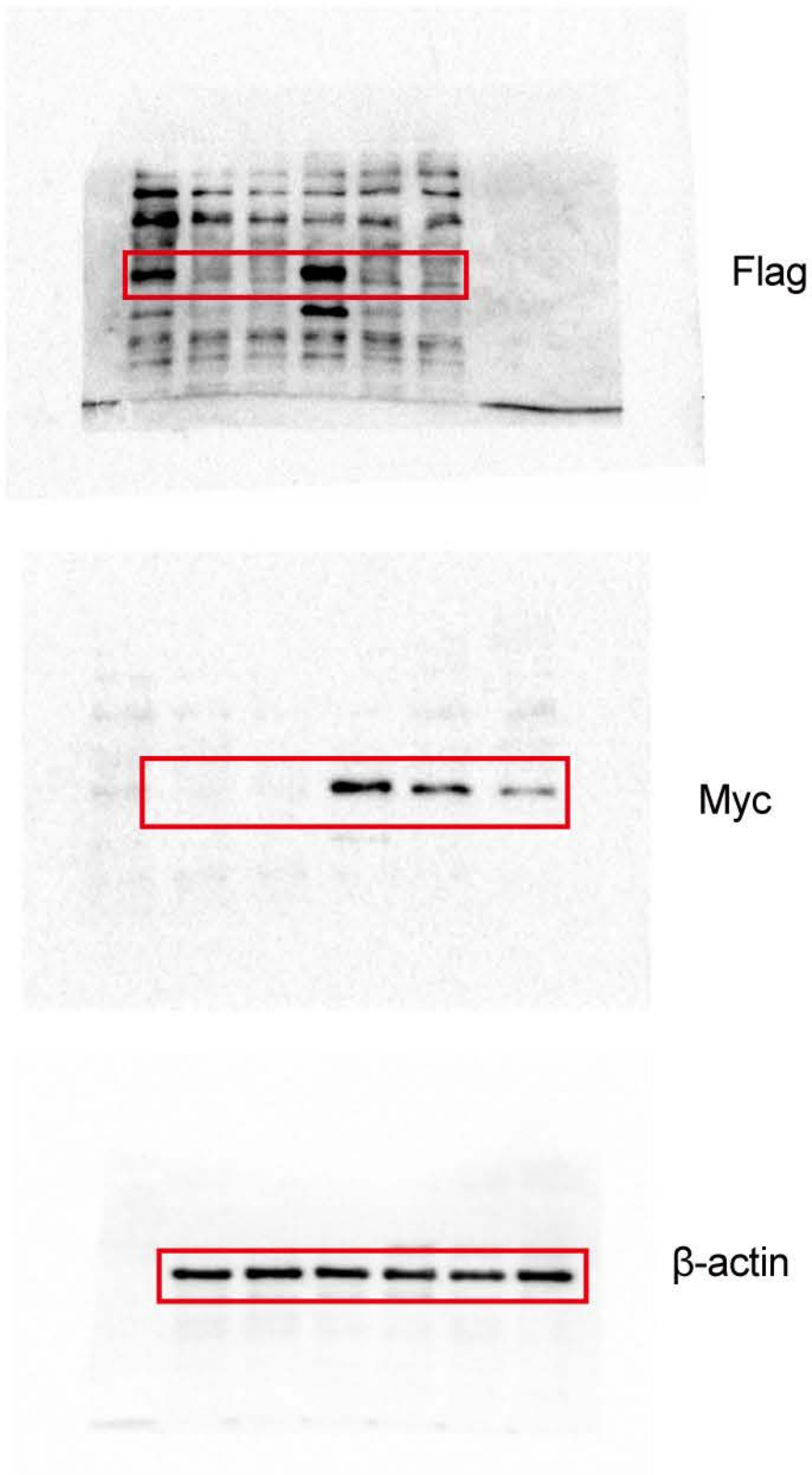
Flag
Myc
β-actin

F4

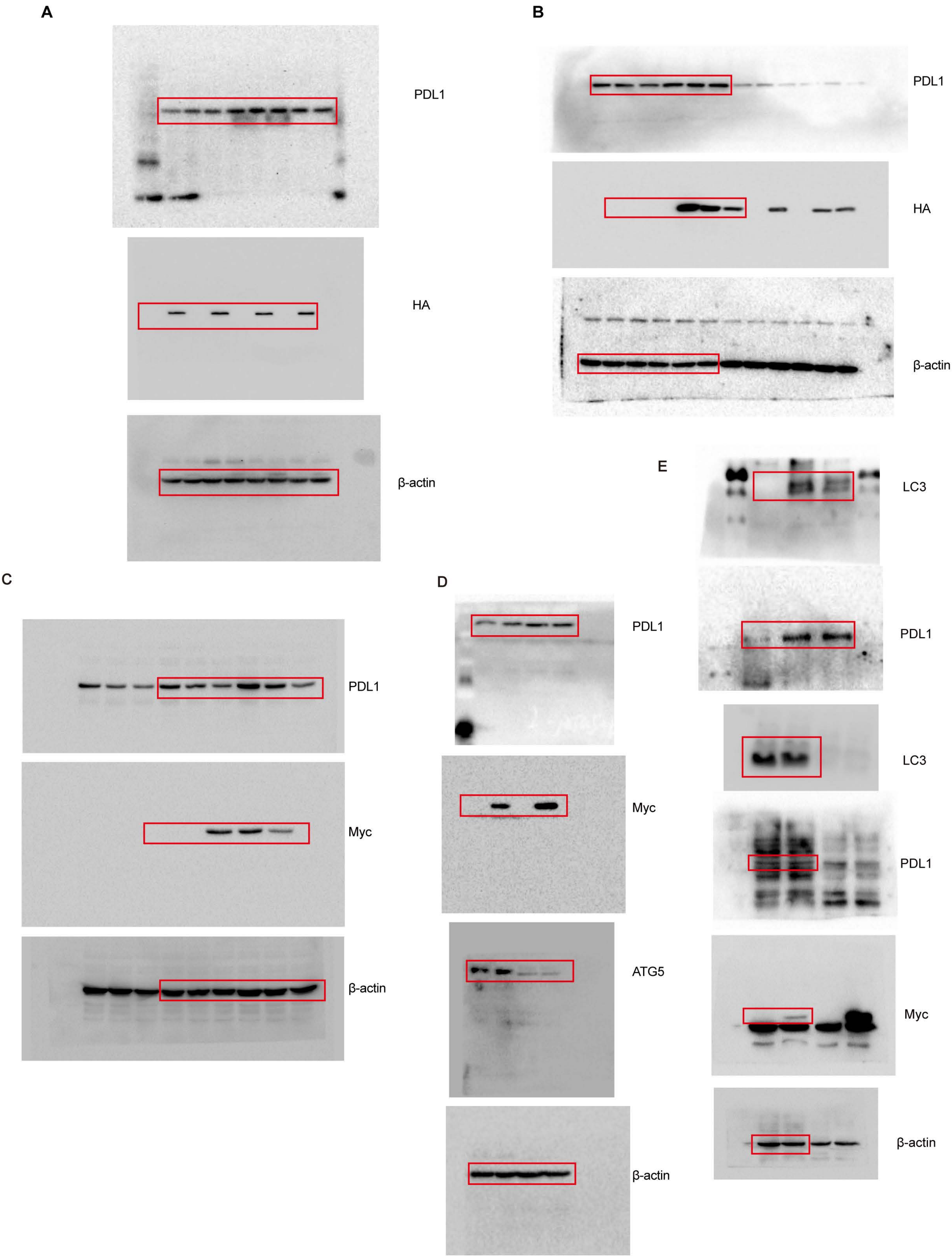
A
PDL1
HA
β-actin
B
PDL1
HA
β-actin
C
PDL1
Myc
β-actin
D
PDL1
Myc
ATG5
β-actin
E
LC3
PDL1
LC3
PDL1
Myc
β-actin

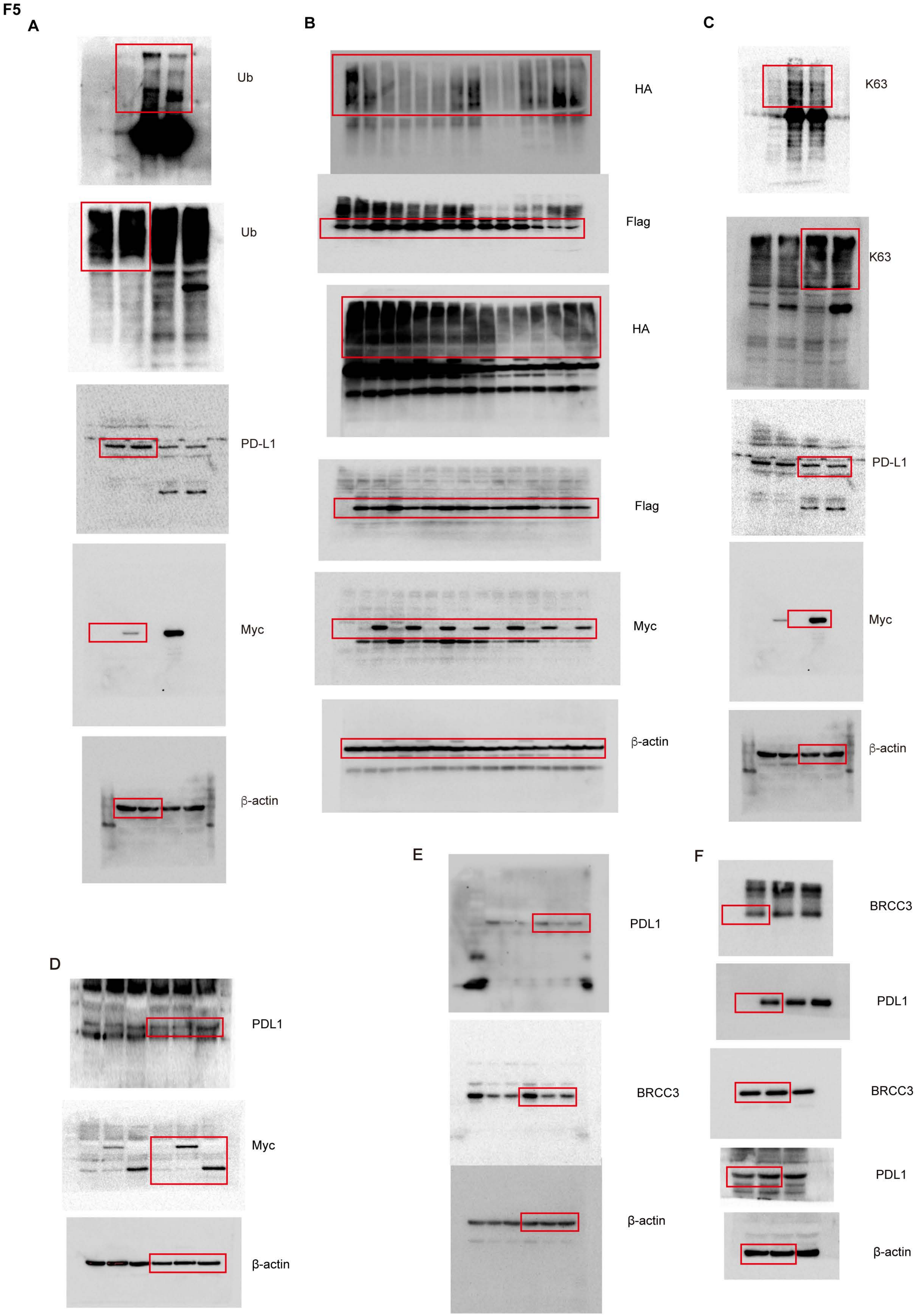
F5
A
Ub
Ub
PD-L1
Myc
β-actin
B
HA
Flag
HA
Flag
Myc
β-actin
C
K63
K63
PD-L1
Myc
β-actin
D
PDL1
Myc
β-actin
E
PDL1
BRCC3
β-actin
F
BRCC3
PDL1
BRCC3
PDL1
β-actin

F5

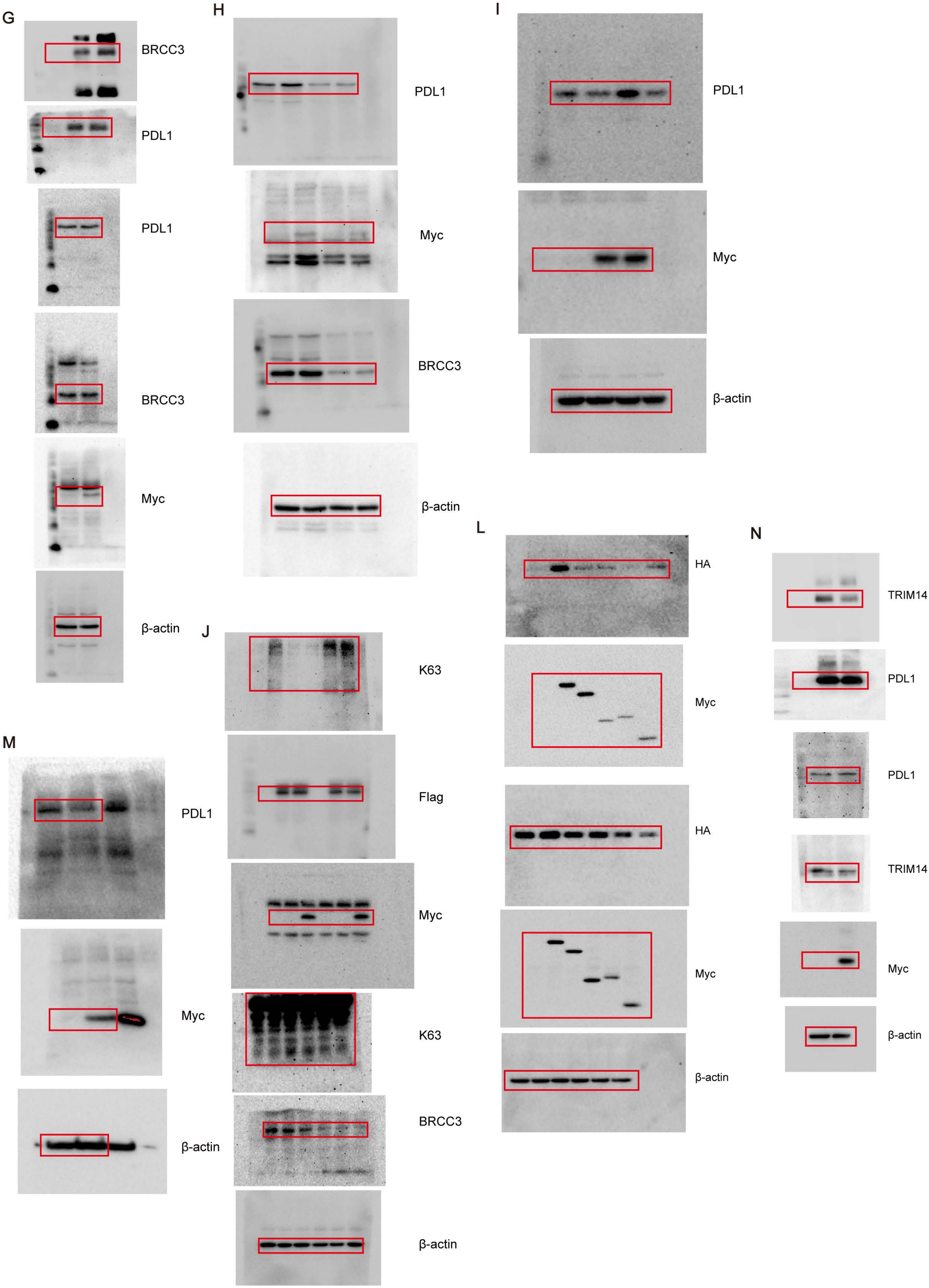
G
BRCC3
PDL1
PDL1
BRCC3
Myc
β-actin
H
PDL1
Myc
BRCC3
β-actin
I
PDL1
Myc
β-actin
J
K63
Flag
Myc
K63
BRCC3
β-actin
L
HA
Myc
HA
Myc
β-actin
M
PDL1
Myc
β-actin
N
TRIM14
PDL1
PDL1
TRIM14
Myc
β-actin
